# From computational high-throughput screenings to the lab: taking metal–organic frameworks out of the computer

**DOI:** 10.1039/d2sc01254e

**Published:** 2022-06-16

**Authors:** Aurelia Li, Rocio Bueno-Perez, David Madden, David Fairen-Jimenez

**Affiliations:** The Adsorption & Advanced Materials Laboratory (A^2^ML), Department of Chemical Engineering & Biotechnology, University of Cambridge Philippa Fawcett Drive Cambridge CB3 0AS UK df334@cam.ac.uk

## Abstract

Metal–organic frameworks (MOFs) are one of the most researched designer materials today, as their high tunability offers scientists a wide space to imagine all kinds of possible structures. Their uniquely flexible customisability spurred the creation of hypothetical datasets and the syntheses of more than 100 000 MOFs officially reported in the Cambridge Structural Database. To scan such large numbers of MOFs, computational high-throughput screenings (HTS) have become the customary method to identify the most promising structure for a given application, and/or to spot useful structure–property relationships. However, despite all these data-mining efforts, only a fraction of HTS studies have identified synthesisable top-performing MOFs that were then further investigated in the lab. In this perspective, we review these specific cases and suggest possible steps to push future HTS more systematically towards synthesisable structures.

## Abstract 2

Computational high-throughput screenings (HTS) have become a standard method of sieving the vast amount of metal–organic frameworks (MOFs) data. But not many HTS studies have been able to bring MOFs to the lab.

## Graphical abstract

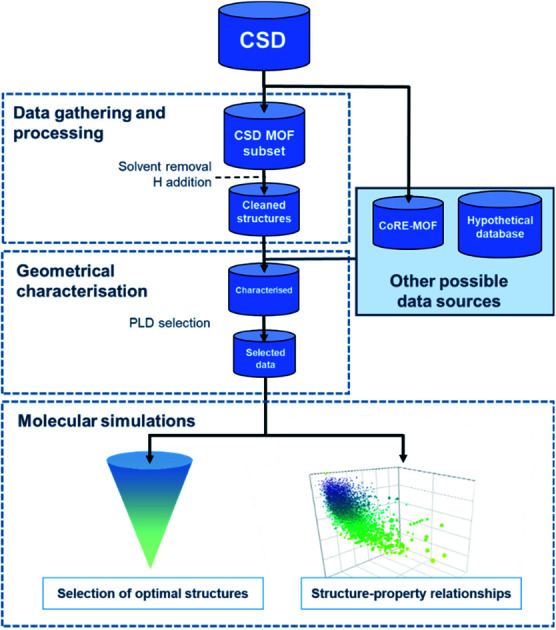

## 1 Introduction

Metal–organic frameworks (MOFs) are a class of crystalline materials assembled from metal atoms or clusters (secondary building units or SBUs) and organic ligands. Their tunability led to the design of structures with a variety of pore sizes, geometries, vast pore volumes and internal surface areas as high as 7800 m^2^ g^−1^.^1^ These extreme porosities can be seen in contrast with those from extremely important materials such as zeolites (1000 m^2^ g^−1^) and activated carbons (3000 m^2^ g^−1^)^2^ and pore volumes. These properties have encouraged researchers to consider MOFs for a wide variety of applications, ranging from gas storage,^3–7^ separation,^8–12^ catalysis^13–15^ to drug delivery^16–20^ and bio-imaging.^17,18,21^ In particular, the ‘building block’ approach^22^ to generating MOFs has encouraged computational and experimental scientists alike to create a large number of hypothetical and experimental structures, the latter reaching almost 100 000 in 2020 in the Cambridge Structural Database (CSD).^23^

With the increasingly large number of structures, computational high-throughput screenings (HTS) have become the standard method to sieve the data. From a small dataset of 14 manually collected MOFs data in 2009 for the study of carbon capture^24^ to half a million structures screened for hydrogen storage in 2019,^25^ a booming number of HTS studies have been published. A quick Google Scholar search with the keywords “computation”, “high-throughput screening” and “metal–organic frameworks” returns 12 800 results. The actual number of relevant HTS studies is likely to be around a few thousand. The aim of these HTS studies are usually two-fold: (i) identify the best performing structure for a given application and (ii) uncover interesting structure–property relationships that can guide researchers towards more rational designs of MOFs in the future. While the attempts at identifying the best structures for a specific task have been numerous,^26^ only a minority of these studies successfully determined *in silico* synthesisable structures that were then taken to the lab for further investigation (*i.e.* at least successfully reproduced a published procedure and compared measured *vs.* calculated material properties, see Table 1). In this perspective, we first review these few cases from both a computational and experimental point of view, thereby highlighting the few applications that have found lab-tested materials. This summary should help future research focus on (i) bringing known best-performing MOFs to the next stage for the applications outlined or (ii) further studying applications that do not have lab-validated candidates yet. We then highlight some challenges the field is faced with when using HTS approaches and suggest the next steps to turn MOFs into industrially viable solutions.

**Table 1:** Computational HTS studies that include experimental synthesis and characterisation of the identified MOFs

Authors	Year	Application	Data	Identified and synthesised MOF
Wilmer *et al.*^27^	2012	Methane storage, 298 K, 35 bar	137 953 hMOFs	NOTT-107
Gómez-Gualdrón *et al.*^38^	2014	Methane storage, 298 K, 5.8–65 bar	Zr-focused 204 ToBACCo MOFs	NU-800
Chung *et al.*^39^	2016	Carbon capture, 313 K, up to 16 bar	Genetic algorithm on 55 163 hMOFs and 5169 CoRE MOFs	NOTT-101/Oet, VEXTUO
Gómez-Gualdrón *et al.*^3^	2016	Hydrogen storage, 77 K, 100 bar 160 K, 5 bar	13 512 ToBACCo MOFs	*she*-MOF-1, NU-1103
Banerjee *et al.*^40^	2016	20 : 80 xenon/krypton	125 000 hMOFs and CoRE MOFs	SBMOF-1
Gee *et al.*^105^	2016	Xylene enrichment, 323 K, 9 bar	4700 CoRE MOFs and a few from RASPA	MIL-47, MIL-125-NH_2_, MIL-140B, MOF-48
Matito-Martos *et al.*^41^	2018	Diethylsulfide (mustard simulant) over water selectivity	2932 DDEC	UTEWOG
Moghadam *et al.*^42^	2018	Oxygen storage, 298 K, 5–140 bar	2932 DDEC	UMCM-152
Boyd *et al.*^43^	2019	15 : 85 CO_2_/N_2_, 298 K, 1 bar 363 K, 0.1 bar	325 000 hMOFs	Al-PMOF, Al-PyrMOF
Bucior *et al.*^44^	2019	Hydrogen storage, 77 K, 100 bar 160 K, 5 bar	A mix of >50 000 including CSD subset	MFU-4L
Ahmed *et al.*^25^	2019	Hydrogen storage, 77 K, 5–100 bar	A mix of 493 458, including CoRE MOFs and the CSD	SNU-70, UMCM-9, PCN-610/NU-100
Rampal *et al.*^45^	2021	CO/N_2_ separation, 298 K, 1–40 bar, 200–298 K, 1 bar, 298–398 K, 1 bar	183 Cu–Cu paddlewheels-containing CoRE MOFs	_mono_HKUST-1
Madden *et al.*^76^	2022	Hydrogen storage, 77 K, 25–50 bar to 160 K, 5 bar	2932 DDEC + 8 benchmark material data from the CSD, RASPA and co-workers	_mono_HKUST-1

## 2 Data sources

The studies presented in Table 1 rely on a variety of databases, which are of two categories: hypothetical or experimental. Hypothetical MOFs are obtained computationally; the hMOF dataset referred to in Table 1 contains 138 956 hypothetical structures built from a “bottom-up” – or Tinkertoy – approach: each structure is generated from the recombination of 102 SBUs and organic linkers of available crystallographic data of existing MOFs.^27^ Since the number and proportion of topologies found in the hMOF dataset were not representing the real space found experimentally, Snurr and co-workers proposed the ToBaCCo (Topology-Based Crystal Constructor) database. ToBaCCo uses a “top-down” (or reverse topological) approach to focus on the diversity of possible MOF topologies.^3^ Here, the number of obtained structures, therefore, varies depending on the chosen topologies – for example, 13 512 unique different MOFs when considering 41 topologies, whereas Boyd *et al.* used a similar approach to generate 300 000 structures from 46 topologies.^28^ While hypothetical structures are disorder-free and readily useable for simulations, their main drawback is the need to find or develop a synthesis method to experimentally validate the computational finding. In contrast, experimental datasets contain structures that have already been synthesised and for which the experimental protocol is known. Indeed, most of the synthesised crystal structures accompanying a publication nowadays are deposited in the curated CSD,^29^ which contains data of experimentally-obtained organic and metal–organic crystal structures in the format of Crystallographic Information Files (CIFs) resulting from X-ray diffraction and similar analyses.^30^ However, due to their experimental nature, the structural data obtained are many times messy and require additional data processing. The Computation-ready, Experimental (CoRE) MOF database was the first publicly available database of existing MOFs. All the structural data were obtained from the CSD and cleaned (solvent removal, addition of missing hydrogen atoms, elimination or repair of disordered structures) so as to be directly ready for computational analysis. As of 2019, the CoRE MOF database was comprised of 14 000 of such curated structures.^31,32^ Building on this, in the density-derived electrostatic and chemical (DDEC)^33,34^ dataset, the partial charges were added to 2900 structures from the CoRE MOF database, allowing the study of adsorption cases where electrostatic interactions play a role. The high quality of the charges and their availability in the CIFs themselves make the DDEC truly fully ready for HTS of multiple gas molecules. Finally, the CSD MOF subset is the first automatically quarterly-updated dataset of MOFs containing almost 100 000 structures as of 2020.^23^ Although by definition it is not computation-ready, it comes with CSD tools for customised, automated, high-throughput cleaning possibilities and solvent removal.^35^ Importantly, this gives the freedom to chose what solvent molecules should be removed, avoiding the removal of crystalline solvent molecules that could be problematic. The choice of one database over another is very likely to impact the result of a study; we here refer the readers to the relevant papers analysing the differences and consequences of such a choice.^36,37^

## 3 The general workflow

**Fig. 1:**
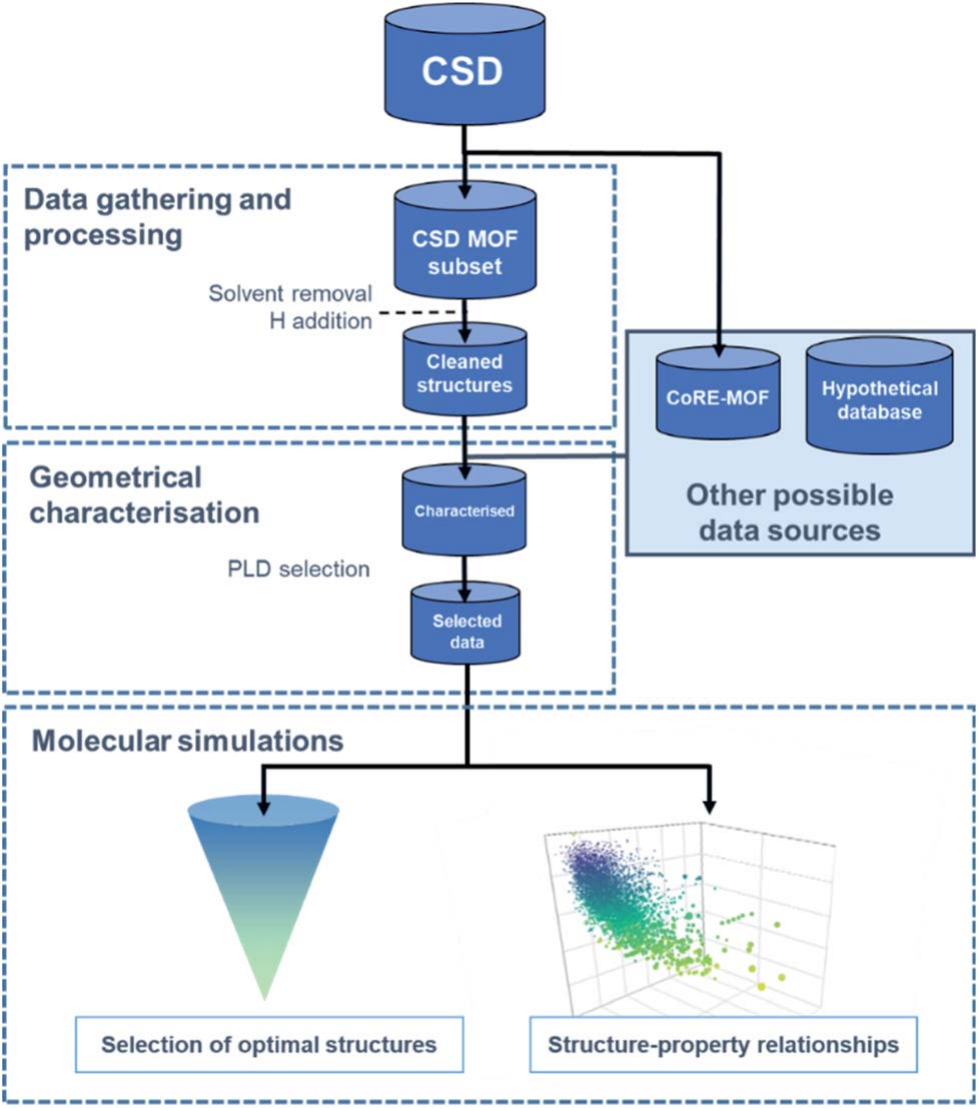
General workflow for the computational high-throughput screening of experimental MOFs.

Regardless of the data source chosen, HTS studies usually follow a similar workflow, as presented in Fig. 1. The first step – structural data gathering and processing – has been briefly described in the previous section. The second step – geometrical characterisation – consists in computing the MOFs' structural descriptors, such as pore-limiting diameter (PLD), largest cavity diameter (LCD), surface area, and pore volume. The PLD is especially useful to eliminate structures for which the gas molecule is too large to travel through, thereby further reducing the number of molecular simulations to run in the next step. In this last step, properties obtained from the simulations and the previous geometrical characterisation can be combined to map out the datasets' structure–property trends, and a small number of top-performing structures can be selected.

### 3.1 Successfully synthesised HTS-identified MOFs

Table 1 presents, in chronological order, several studies that have successfully brought HTS-identified MOFs out of the computer and reproduced the relevant synthesis for further characterisation and validation in the lab. These studies are reviewed case by case in the following paragraphs, highlighting the main results and conclusions.

### 3.2 Methane storage

Wilmer *et al.* carried out one of the earliest high-throughput studies for the adsorption of methane at 298 K and 35 bar.^27^ The storage of methane at room temperature and high pressures is extremely useful for natural gas-powered vehicles, the main challenge being the ability to store enough methane for given driving distances. MOFs could potentially lead to cheaper, high-density tanks that meet the US Department of Energy (DoE) target of 180 cm^3^ (STP) cm^−3^ at 298 K and 35 bars. For this screening, the authors used their in-house hMOFs database and used several rounds of GCMC simulations with an increased number of cycles on a smaller amount of data, with the best-performing data after each round. Among the 300 top structures that performed better than the then world-record (230 cm^3^ (STP) cm^−3^), the existing – but unbeknownst to the authors – Cu–Cu paddle-wheel-based NOTT-107 was synthesised. However, the measured uptake was 8% lower than the predicted value and lower than the record. The authors explained the disparity with the possible incomplete pore activation of the synthesised MOF. In addition, the authors found a trade-off between maximising the structures' gravimetric surface area and their storage capability, with an optimum point at 2500–3000 m^2^ g^−1^. A large surface area or pore volume that is too large also has a negative impact on the density and the uptake.^46^ In fact, they found that the ideal pore size corresponded to either exactly one or two methane molecules. The authors also found that methyl-functionalised MOFs, such as the identified NOTT-107, usually performed better.

Knowing the outstanding stability of zirconium MOFs, Gómez Gualdrón *et al.* generated 204 hypothetical ToBaCCo MOFs based on four topologies compatible with zirconium MOFs. The GCMC simulations performed at room temperature and 5.8–65 bar pressure swing revealed the top-performing structure – later on named as NU-800. As expected, and as explained previously, identifying the best hypothetical structure required additional effort to develop a synthesis. The measured deliverable capacity of NU-800 was 10% lower than the simulated, 167 *vs.* 187 cm^3^ (STP) cm^−3^, respectively, which places NU-800 among the honourable mentions but still far behind the DoE target of 263 cm^3^ (STP) cm^−3^*deliverable* capacity under these conditions. The lower capacity was once again attributed to the incomplete pore activation of the structure. The advantage of such a hypothetical database, however, is the ability to compare apples to apples. For example: by examining zirconium MOFs formed with the same topology but varying linker isomerism, the authors concluded that the best packing is obtained when the alkyne groups – instead of the phenyl rings – are close to the zirconium nodes.

### 3.3 Carbon capture

Chung *et al.* developed a genetic algorithm (GA) to identify top-performing structures for the capture of carbon at 313 K at a lower computational cost.^39^ Carbon capture and storage represents an interesting transitional solution while fossil fuels are still in use. For recent power plants, carbon can be captured *via* a precombustion carbon technology, where natural gas is first reformed into a mixture of CO and H_2_, before going through a water–gas shift reaction which produces high-pressure steam of CO_2_ and H_2_. The final carbon is obtained by removing it from the stream. In terms of their simulation methods, GAs are a class of optimisation methods inspired by the theory of natural selection. The algorithm starts with an initial population of structures and a definition of a fitness function. The genetically fittest structures then evolve to give birth to the subsequent generations. In this case, instead of performing brute-force GCMC simulations on a subset of 55 163 hMOFs, the authors calculated working capacities, selectivities and adsorbent performance scores only on structures that were deemed the fittest by the GA, thus reducing the computational time by two orders of magnitude. The properties of the fittest hMOFs were then studied to look for promising structures in the CoRE MOF database. The hypothetical ethoxy-functionalised NOTT-101 was found to be the best performing MOF. After applying the GA to the CoRE MOF database, the structure with the CSD refcode VEXTUO was found to be another promising structure. Both structures were synthesised and NOTT-101/Oet was confirmed as the new record for this application. Similarly, Boyd *et al.* used their in-house 325 000 hypothetical database to identify the most relevant binding sites – or “adsorbaphores” – for CO_2_/N_2_ separation.^43^ The authors defined “adsorbaphore” here as the “common pore shape and chemistry of a binding site in a MOF that provides optimal interactions to preferentially bind to a particular guest molecule”. From the top-ranked 8325 materials, they identified 106 680 adsorbaphores that were then classified into three categories: (A1) those with two parallel aromatic rings 7 Å apart, (A2) those composed of metal–oxygen–metal bridges and (A3) open metal sites. Among these, the first group of adsorbaphores (A1) were found to be less H_2_O-binding. The authors then chose a topology in which such binding sites can be found or tuned. As the *frz* topology is an experimentally sound choice, they generated 35 such isoreticular MOFs and computationally confirmed their CO_2_/N_2_ selectivity at low pressures as well as the low influence of humidity. At higher partial pressures of water, however, H-bond formation tends to dominate. When this is not the case, the H-bonds are frustrated by the pore shape. Based on this, two structures – Al-PMOF and Al-PyrMOF – were then synthesised. The measured isotherms matched those predicted, and further breakthrough experiments confirmed that humidity had little influence on their performance. Although the materials synthesised do not have the highest reported CO_2_ working capacity, Al-PMOF outperforms the commercially used zeolite 13× and activated carbon.

### 3.4 CO/N_2_ separation

Carbon monoxide is a key raw material in the chemical industry. One major application is the production of acetic acid *via* the CATIVA process, which uses carbon monoxide and methanol as feedstocks.^47^ However, whilst methanol is easy to obtain, carbon monoxide must be produced locally. The current technologies produce carbon monoxide by purifying syngas (a mixture of CO, H_2_, N_2_ and CH_4_ mainly), but CO/N_2_ is particularly difficult to separate due to their similar physical properties. Based on previous studies showing that Cu–Cu paddlewheels favour CO separations,^48–50^ Rampal *et al.* selected a subset of 183 Cu–Cu paddle-wheel structures from CoRE MOF, on which they ran GCMC simulations combined with three sets of process modelling.^45^ The latter consisted of the simulation of a 3-steps pressure-swing adsorption (PSA) simulation at 298 K and 1–40 bar, and two 3-steps temperature-swing adsorption simulation (TSA), one at 1 bar and 200–298 K, and another at 1 bar, 298–398 K. The analysis of the uptakes obtained from the GCMC simulations and the added metrics of purity, recovery, and amount of product generated per unit of mass adsorbent calculated from the process simulations, led to the selection of four candidates. Upon further analysis of PSA performance, HKUST-1 was synthesised in powder and as a densified monolith form (_mono_HKUST-1).^51^ The measured performance of both forms accurately matched the calculated outcome, with _mono_HKUST-1 having the additional advantage of being in an industry-friendly pelletised form.

### 3.5 Xenon/krypton separation

Xenon/krypton separation is of great industrial interest. As rare gases, they both exist in low concentrations in nature. Xenon is found at 0.087 parts per million by volume (ppmv) in the atmosphere, and krypton at 1.14 ppmv.^52^ Yet, both play important roles in applications ranging from medical imaging^53^ to anaesthetics,^53,54^ and from lighting,^55^ lasers^56^ to double-glazing^56^ and satellite propellants.^57^ Currently, a 20 : 80 mixture of xenon/krypton is first obtained as a byproduct of cryogenic distillations for the separation of oxygen and nitrogen in the air.^58,59^ Additional cryogenic technologies are then required to obtain pure xenon and krypton. The low concentrations mean the price of high-purity xenon is currently as high as 5000 USD per kilogram.^55^ Selective adsorption in porous materials could be a potential cheaper alternative. Banerjee *et al.* screened 125 000 hypothetical and experimental MOFs and identified SBMOF-1 ^5^ to be the top-performer for xenon/krypton separation at 298 K and 1 bar.^40^ SBMOF-1 is an experimental MOF that had been previously identified, albeit only computationally.^58^ The measured isotherm only matched the prediction at low pressure when the structure was activated in low temperature, but the overall experimental results were very positive. The relatively lower surface area of SBMOF-1 means its saturation loading is lower compared to its peers, but it has the highest reported selectivity for xenon, a fast saturation uptake, robustness to multiple adsorption–desorption cycles as well as to humidity.

### 3.6 Xylene enrichment

Xylene isomers (*p*-xylene, *o*-xylene, *m*-xylene and ethylbenzene) are often used as industrial solvents or chemical intermediates. However, they usually come as a mix, and their separation is tricky because their boiling points are close. Current methods to recover one of these isomers are crystallisation or simulated moving bed processes. Here, Gee *et al.* investigated the use of MOFs for the separation of a 0.33 : 1:2 : 1 mixture of ethylbenzene/*o*-xylene/*m*-xylene/*p*-xylene at 9 bar and 323 K.^105^ In particular, they targeted the recovery of *p*-xylene, used in the synthesis of terephthalic acid. From the HTS of 4700 CoRE MOF structures, complemented with MOFs available in the software RASPA,^60^ they identified four MOFs that are chemically and thermally stable and synthesisable with commercially available ligands: MIL-47 (*o*-xylene selective), MIL-125-NH_2_, MIL-140B and MOF-48. The latter two were found to have selectivities higher than the state-of-the-art zeolite BaX currently used in the industry. The selectivity and capacity of MOF-48 could be also increased by further optimising its synthesis and activation procedure.

### 3.7 Capture of chemical warfare agents

The use of molecular simulations for the capture of chemical warfare agents (CWAs) has a clear advantage over experimental work. Whereas experimental work in most labs is restricted to the use of simulants, molecular simulations do not have these safety concerns or limitations. Using the DDEC database, Matitos-Martos *et al.* explored a range CWAs and simulants, finding an ideal structure for the capture of diethylsulfide (DES) in moist environments. DES is a simulant of the CWA mustard gas. A difficulty here is that, in this application, one needs to have a hydrophobic MOF where water will not compete. However, water isotherms are expensive in terms of simulation time. To solve this issue, they followed the approach described by Moghadam *et al.*,^61^ running first a round of preliminary selection using the water Henry's constants to estimate the structures' hydrophobicity and using ZIF-8 as a hydrophobic benchmark. The Henry's constants were obtained using Widom test particle insertion methods^62^ – something that can reduce the equilibration time several orders of magnitude – and were deemed a good indication of the adsorbent–adsorbate interactions. By running GCMC simulations on 183 selected, hydrophobic MOFs, they found that the highest chemical warfare agent-MOF interactions took place in structures with rather high surface areas (up to 2000 m^2^ g^−1^) and with an optimum Henry's constant for LCDs between 5 and 6 Å.^39^ The identified structure, of CSD refcode UTEWOG, was synthesised according to the existing protocol and its performance in humid conditions validated. Importantly, they also found an excellent correlation between the performance of a specific CWA with the other CWAs and simulants studied.

### 3.8 Oxygen storage

Oxygen storage is a relatively less explored gas adsorption application with MOFs; there are also safety concerns when using high-pressure oxygen experimentally. Its potential uses include improved oxygen tanks in the healthcare industry as first aiders, in the military and aerospace industries.^63^ Using again the DDEC database, Moghadam *et al.* performed GCMC simulations and found the best existing candidate for oxygen storage at 298 K and a pressure swing of 5–140 bars, UMCM-152.^42^ The identified structure was then synthesised and its uptake was confirmed experimentally to be 22.5% higher than the previously best-performing structure reported in the literature. This study also advanced in the statistical analysis of the obtained data. More importantly, it provided a new dynamic visualisation software (described in more detail in Section 4.1) to analyse trade-offs between maximising surface area, and other textural properties, and storage capabilities. In this case, they found a ceiling of 250 cm^3^ (STP) cm^−3^ for oxygen storage. Importantly, structures with cavities larger than 10 Å and void fractions higher than 0.8 did not improve this volumetric uptake.

### 3.9 Hydrogen storage

As a promising clean vehicular fuel, hydrogen is by far the most computationally studied gas for adsorption application in MOFs.^3,64–75^ Gómez Gualdrón *et al.* screened their ToBaCCo database for hydrogen adsorption under the temperature and pressure swing (TPS) conditions of 77 K, 100 bar to 160 K, 5 bar.^3^ Of the 13 512 structures screened, some of the best-performing ones had already been synthesised. However, NU-1103 had not been experimentally tested at the chosen conditions, and its working capacity was later on confirmed to be 43.2 g L^−1^, surpassing the target of 30 g L^−1^ set by the US Department of Energy (DoE) for 2020.^76^ It is important to highlight that the volumetric capacities included here are obtained using the theoretical single-crystal densities of the MOFs and do not take into account any packing issues. To further demonstrate the potential of hypothetical databases in widening the known topology landscape of MOFs, the authors chose to focus on the rarely encountered *she* topology. The latter is particularly interesting as it is not prone to interpenetration. Of the 50 *she*-MOFs generated, four top-performing structures were synthesised. Of these, only *she*-MOF-1 was considered for hydrogen adsorption measurements, and its working capacity was determined to be 43.4 g L^−1^. However, the authors indicated that its low stability might be a drawback for any industrial application. More recently, Bucior *et al.* combined GCMC and supervised learning based on the structures' potential energy histograms to screen a dataset of more than 50 000 structures composed of a mix of different available experimental databases for the same hydrogen storage conditions.^44^ In this study, the authors found that a relatively weak adsorbate-MOF interaction is ideal for hydrogen storage at cryogenic conditions, and identified MFU-4L as one of the top-performing materials with an experimental deliverable capacity of 47 g L^−1^, thus ranking among other previously identified structures.^75^ Ahmed *et al.* soon after screened *ca.* 500 000 structures composed of a mix of all available hypothetical and experimental data for hydrogen storage at the cryogenic pressure swing conditions of 5–100 bar. After a first selection of structures using the semi-empirical Chahine rule, GCMC was applied to *ca.* 44 000 structures. Three candidates were identified: SNU-70, UMCM-9, PCN-610/NU-100, all of which were synthesised and shown to perform better than MFU-4L at the same previous TPS conditions. PCN-610/NU-100 and UMCM-9 were existing MOFs whereas SNU-70 was a hypothetical one. Madden *et al.* screened the DDEC database, to which they added 8 benchmark material data from the CSD, RASPA^60^ and co-workers, at 5, 25, 50, 100 bar and 77, 160, 198, 233 and 298 K.^77^ They investigated purely cryogenic delivery conditions (25, 50 and 100 bar/77 K to 5 bar/160 K) and near-ambient delivery conditions (100 bar/198 K and 100 bar/233 K to 5 bar/298 K). The data analysis showed that structures (such as MOF-5, IRMOF-20, NU-1500-Al, IRMOF-10, NU-1501-Al) with high surface areas and large pore volumes present higher deliverable capacities at low temperatures and high pressures. However, denser structures with stronger adsorbated-adsorbent interactions (such as HKUST-1 and Ni_2_(dobdc)) present higher deliverable capacities at higher temperatures and lower pressures. A further principal component analysis revealed the importance of optimising a material's density when choosing the adsorption pressure, and the existence of an upper threshold for the adsorption pressure around 50–55 bar. Beyond this range, the performance of the materials starts to deteriorate. Due to the commercially available ligand, its ease of synthesis and high density, _mono_HKUST-1 was synthesised and proven to deliver 41 and 42 g L^−1^ at 25 and 50 bar, respectively, when used in the TPS condition of 25–50 bar/77 K to 5 bar/160 K. This corresponds to an 80% decrease of the operating pressure when compared to benchmark materials, and 83% compared to compressed H_2_ gas.

## 4 What next?

Validating the lab-scale feasibility of MOFs found *in silico* is only the first of many steps to bring the material to an industrially useable stage. And yet, as shown in the previous section, only a minority of published HTS studies have led to experimental testing. While any computational finding is valuable to the community, corroborating the results is important to (i) validate the HTS process and (ii) pave the ground for the next research steps. Structures that are proven to be synthesisable at a reasonable cost are more likely to be considered for further system integration. There are many possible reasons for such few experiments-backed HTS studies, such as the lack of human resources or laboratory equipment, expensive reagents or difficult – or even unreproducible – synthesis protocols. In this perspective, we discuss (i) the importance of bridging the communication gap between computational and experimental researchers to foster collaboration, (ii) the need to move towards a holistic HTS approach taking into account synthesis metrics and (iii) the role of digitalisation in improving reproducibility. While some of these issues are not generic to the MOF field, they are exacerbated by the sheer number of MOF structural data available and produced each year. In fact, many of the directions of improvement implemented in the past and presented here are inspired by pioneering work done in data-heavy fields such as bioinformatics and other materials fields. We discuss here how these elements can be specifically incorporated into the MOF HTS context to accelerate the identification and testing of MOFs.

### 4.1 Fostering computational–experimental collaboration with better data communication

**Fig. 2:**
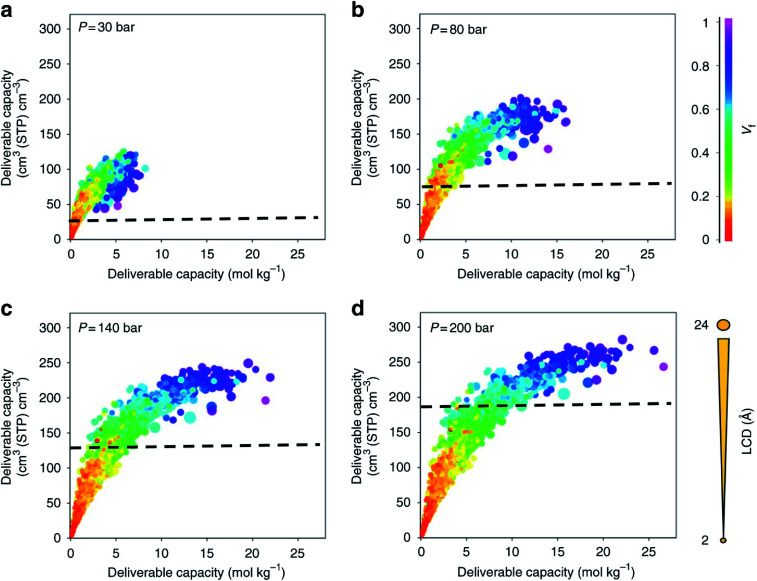
Visualisation of the structure–property relationships for oxygen storage in MOFs by Moghadam *et al.*^42^ Oxygen volumetric and gravimetric deliverable capacity is plotted *vs.* the largest cavity diameter (LCD) and void fraction (*V*_f_) for 2932 MOF structures at (a) 30 bar, (b) 80 bar, (c) 140 bar and (d) 200 bar storage pressures and 298 K. The release pressure is kept fixed at 5 bar for all storage pressures. The dashed lines mark the amount of oxygen adsorbed in an empty tank. Each point in the graph represents a different structure. The data points are colour coded and sized according to *V*_f_ and LCD, respectively. All the plots can be visualised on a multidimensional interactive web app available at https://aam.ceb.cam.ac.uk/mof-explorer. However, only the rainbow gradient is available for the colour axis. Reproduced from *Nat Commun.*, **9**, 1378 (2018) with permission from Springer Nature.

With a large amount of data comes the following questions: what to visualise, how to best visualise it, and how to share it. While the topic of data visualisation might seem trivial, the clear, flexible, informative, biased or un-biased presentation of data is crucial for (i) conveying the desired message to the entire community – computational but also experimental – and (ii) carrying out extensive exploratory data analyses prior to applying the plethora of now ubiquitous machine learning algorithms. And it seems that scientists are not the best at creating visualisations just yet,^78^ so much so that *Nature Methods* published a set of guidelines from picking the right plot for the right data, to using colourblind-friendly colours, from avoiding rainbow gradients for continuous data to choosing the right fonts.^79–83^ Some remarkable improvements have however been made recently in the field. Along with the (re)discovery of UMCM-152, and being inspired by the work from Rosling *et al.* on Gapminder,^84^ Moghadam *et al.* published an online interactive data explorer where users can plot all the available textural and adsorption properties in order to spot interesting and additional structure–property trends at multiple pressures and potential structures of interest.^42^ Over 1000 plots can be easily obtained, by choosing different axes, colours and sizes for the available variables. Users can also follow the evolution of the properties of a structure as the pressure point changes. In addition, each structure has a link to the corresponding CSD entry web page. Plots can be zoomed in and out, the corresponding data filtered *a priori* or *a posteriori* and snapshots can be extracted directly. Fig. 2 shows snapshots of the plots that can be obtained with this webtool. Such data visualisation tools were then adopted by Matito-Martos *et al.* and others for the publication of the data obtained.^41^

There is still, however, a gap between the users being able to visualise other people's data and plotting their own. In a *Nature* toolbox section, Perkel called for more accessible data visualisation tools.^85^ In particular, the ability for researchers to easily plot interactive figures could not only drive story-telling but also reproducibility. Following up on this, Balzer *et al.* recently developed Wiz, a free web app for the codeless, interactive visualisation of any large datasets.^86^ This tool, born from the MOF field, is announced to extend its functionalities to data analysis. Recently, Sarkisov *et al.* published an online software for the computation of principal component analysis for MOFs with pre-tabulated data.^87^ This tool is planned to accommodate any kind of data, thus paving the way to lowering the entry barrier to big data analysis.

**Fig. 3:**
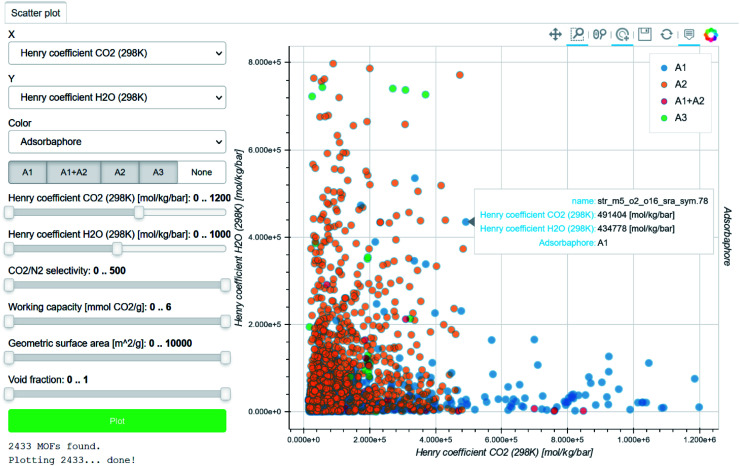
Screenshot of a Materials Cloud^90^ interactive visualisation of data computed by Boyd *et al.* for the identification of top-performing materials for wet flue gas carbon capture.^43^ Each point corresponds to a structure, for which the name and plotted information are accessible by hovering the cursor on it. The H_2_O Henry coefficients are plotted against the CO_2_ Henry coefficients. The points are colour-coded according to the three types of adsorbaphores identified: (A1) those with two parallel aromatic rings 7 Å apart, (A2) those composed of metal–oxygen–metal bridges and (A3) open metal sites.

Beyond plotting and visualising the data, the easy sharing and tracking of data are crucial for HTS studies. Pizzi *et al.* introduced in 2015 the Automated Interactive Infrastructure and Database (AiiDA) for Computational Science to help computational scientists manage the various workflows involved in handling a large amount of data.^88,89^ The outcome of each study and the relevant interactive data visualisations are automatically collated in the Materials Cloud platform (Fig. 3).^90^ Coudert also noted other similar initiatives in gathering data calculated during different studies using different databases.^91^ He highlighted the still much-needed efforts to create open databases that follow the FAIR principle (findable, accessible, interoperable, reusable). However, most of these efforts remain within the computational community. As discussed by Coudert, it is now important to link the calculated data to experimental data.^91^ The latter could be scraped from the existing literature or added as further HTS studies are validated, for instance. We believe that interlinking computational data with experimental data will move the MOF community further towards a knowledge base.

### 4.2 Towards a holistic HTS framework

**Fig. 4:**
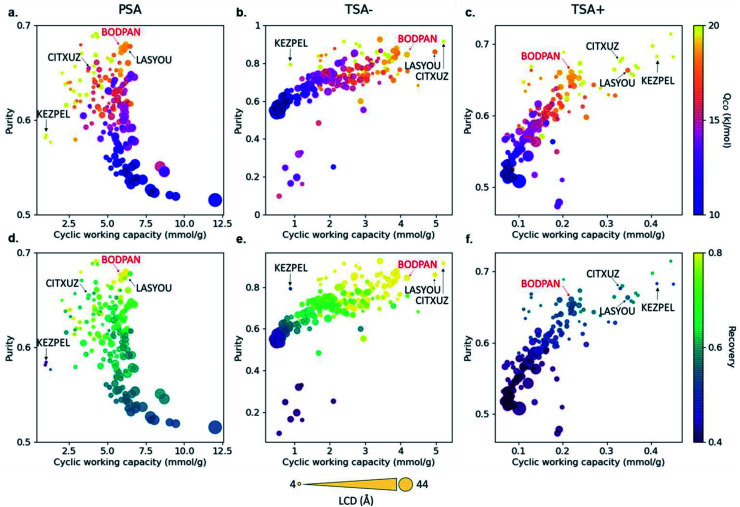
Visualisation of structure–process relationships obtained from the process simulations of 183 MOFs for the CO/N_2_ separation by Rampal et al.^45^ Purity *vs.* cyclic working capacity is plotted for PSA, TSA^−^ and TSA^+^ processes, where the color scale represents (a–c) the CO heat of adsorption and (d–f) the recovery. Symbol size represents the largest cavity diameter (LCD) in Å. Four structures with top performance are named and highlighted, including HKUST-1 (BODPAN), labeled in red. PSA conditions are 298 K, with adsorption at 40 bar and desorption at 1 bar; TSA^−^ conditions are 1 bar, with adsorption at 200 K and desorption at 298 K; TSA^+^ conditions are 1 bar, with adsorption at 298 K and desorption at 398 K. All the plots can be visualised on a multidimensional interactive web app available at https://aam.ceb.cam.ac.uk/mofexplorer.html. Reproduced from Chem. Sci., 2021, **12**, 12068–12081 with permission from the Royal Society of Chemistry.

Most HTS studies focus on identifying top-performing structures based on only a few metrics, such as volumetric and gravimetric uptakes, selectivities and geometrical properties. However, to be useful at an industrial scale, MOFs need to be integrated into broader systems that have their own constraints. These bring a new – large – set of conditions that the materials need to satisfy. The CO/N_2_ example from Rampal *et al.* showed the inclusion of indicators specific to the processes considered (Fig. 4).^45^ But beyond process simulations, another major industrial constraint is cost. While it might be difficult to accurately estimate the economics of a final MOF-system, some additional data can be included early on in the HTS, such as reagents costs, equipment needed (at a lab scale first) and associated costs, estimated overall synthesis time needed and estimated human time needed. These indicators, either included as standalone measures or combined into a new feasibility metric, can help discard any structure that would be too costly or difficult to produce. All this data is already available, albeit scattered across the web. For structures that have been synthesised, the original papers contain the procedures, and, therefore, the reagents needed and synthesis steps. In fact, Park *et al.* very recently extracted synthesis protocols by applying natural language processing on 47 187 papers from the CSD.^92^ The mined information included the precursors, solvents and various synthesis conditions. The next step would be to connect the reagents to their costs, either by connecting the relevant databases or by scraping the web. Adding feasibility metrics to a comprehensive database – such as a computational–experimental knowledge base – would be very useful to the MOF community. The extracted procedures can also help predict the experimental protocols for hypothetical MOFs, as demonstrated by Luo *et al.*^93^ In their work, a database (SynMOF) containing 983 structures and scraped protocols from the CSD was used to train different regression algorithms and to predict synthetic conditions for a given structure. After comparison with 11 expert chemists' intuition, it was found that the algorithm picked up patterns among the data that were new to the human scientists.

### 4.3 Improving reproducibility

One major issue when it comes to synthesising a structure following a procedure written by another lab is its reproducibility. From one lab to another, many things can change and affect the synthesis: lab equipment, reagents providers, and product batches to name a few, but also – and mostly – human intervention and its less trackable impact. This means that even if a structure is synthesised, it might behave differently from the original report. This is exemplified by Sholl *et al.* who studied the reproducibility of measured CO_2_ isotherms listed in the NIST/ARPA-E Adsorption Database.^94^ In total, 211 measured isotherms in 27 different MOFs were analysed. Among these isotherms, only a few were reproducible and 20% were actually outliers and thus should not be used to draw any conclusions about the materials. One way to improve reproducibility here is to establish reference isotherms for reference materials. Such initiatives already exist in the zeolites field, where independent laboratories were tasked with the isotherm measurement of a sample from the same reference material.^95^ More surprisingly, the Brunauer–Emmett–Teller (BET) areas calculated from the same raw adsorption isotherm can also be challenging to reproduce, as shown by Osterrieth *et al.*^96^ This is not only due to the difficulty of correctly applying common systematic procedures, such as the Rouquerol criteria, but to the necessity to expand them. To prove this, the authors asked 61 different laboratories to determine the BET areas of 18 measured isotherms of micro- and mesoporous materials and almost no two groups obtained the same values, with a spread of at least 300 m^2^ g^−1^ (for an 833 m^2^ g^−1^ zeolite) and as high as 7584 m^2^ g^−1^ (for a 5684 m^2^ g^−1^ MOF). One way to avoid such reproducibility issues is digitalisation. To solve the question of the BET area calculation, Osterrieth *et al.* developed a software, the BET surface identification, or BETSI, to unambiguously determine BET areas from a given isotherm in a standardised manner. In the lab, digitalisation also means automation. Although systematic synthesis has been explored previously by Stock and co-workers^97,98^ and Yaghi and co-workers,^99^ this field has broad possibilities when including robotics. Not only can machines minimise human biases in the steps where they are introduced, but they also save scientists from time-consuming, repetitive tasks. This is all the more true when it comes to optimising an experimental procedure, where only one variable is changed at a time. Robots are particularly helpful in these situations, as they can be programmed to explore chemical spaces that would take human scientists an incomparable longer time to achieve. Even more time and resources can be saved in the long run if the robots are equipped with an active learning brain, where it chooses the next condition to test based on learned data, thus closing the loop of scientific discovery. The combination of automated high-throughput experiments and artificial intelligence in the lab is not new. Indeed, King *et al.* introduced the concept of ‘Robot Scientist’ in 2009, with their robot ‘Adam’ who autonomously tested its own hypotheses.^100^ However, most of the robots developed since then remained static and could not cater for the complexity and variety of experiments required in a chemistry lab. In addition, setting up such a robot took significant time and effort; ‘Adam’ was born after a 7 year long process, for instance.^101^

The development in 2020 of a *mobile* robot chemist by Burger *et al.* changed the game.^102^ This time, the modularity introduced means the same robot can be more easily tailored to another lab space with different operations and equipment, and the set-up time was reduced significantly. While it took Burger *et al.* two years to set up theirs, it is estimated that transferring the same robot using the pre-developed protocols and software should take less time.^102^ Still, adapting the robots' brains to a completely different experimental goal is not straightforward. To help tune a robotic platform, Cronin and co-workers developed ‘compiler’ – a program that translates experimental procedures into instructions for the robot.^103^ Importantly, the synthetic protocols are codified with a chemical programming language based on a universal and interoperable standard, meaning that any procedure can be converted to a shareable code, and thus guaranteed to be reproducible. Although such significant digitalisation is not within every lab's reach, small improvements can still be made, such as switching to electronic lab notebooks to track experimental procedures or sharing “failed” syntheses – *e.g.* in a computational–experimental knowledge base. “Negative” results not only prevent other chemists from wasting time and resources but also provide computational scientists with valuable data on which to train machine learning algorithms for the prediction of synthesis conditions.^104^

## 5 Outlook

**Fig. 5:**
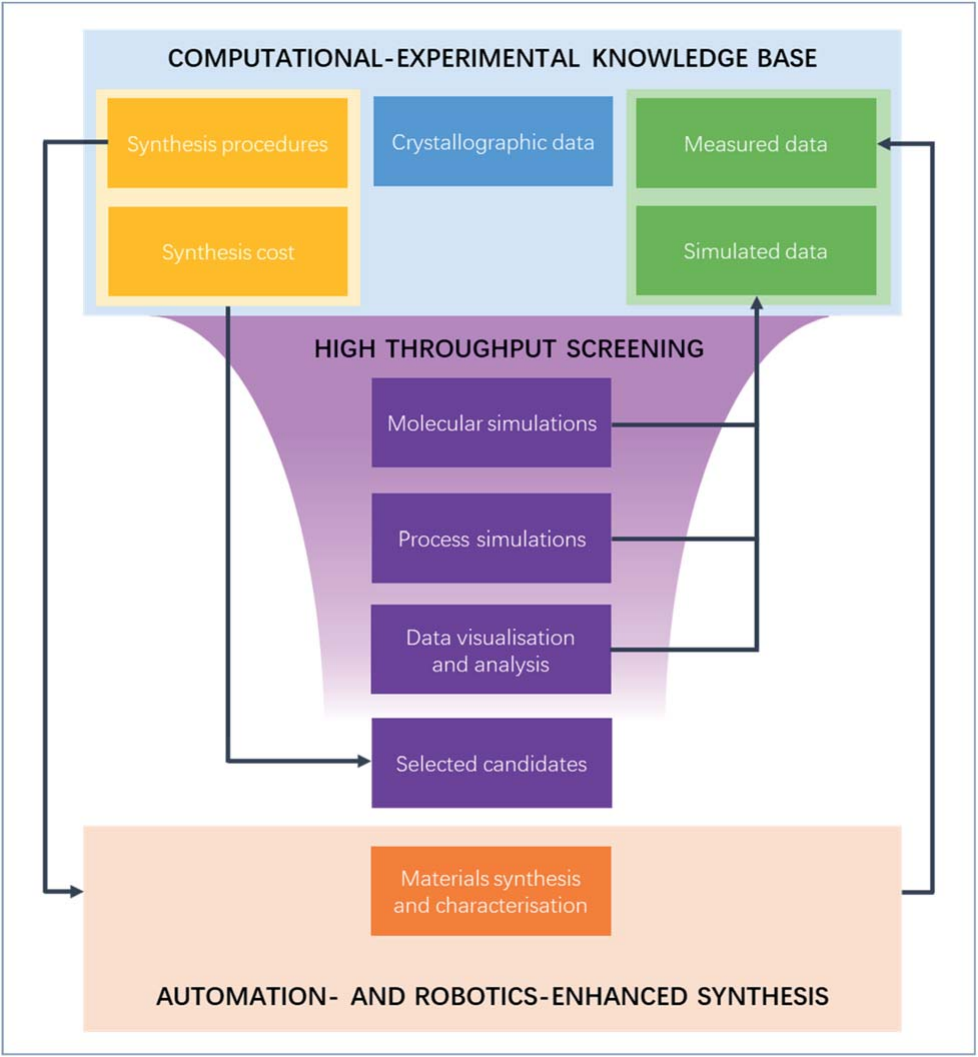
Digitalisation of the high-throughput screening-assisted MOF discovery workflow. A closed loop between a common computational–experimental knowledge base and automation- and robotics-enhanced syntheses.

With the increasing number of MOFs synthesised, the computational MOF community has gradually moved since the early 2010s to HTS to find the perfect needles in the haystack. While HTS has now become a relatively standard procedure to identify interesting structures, only a few have actually led to the discovery of top-performing materials that were brought out of the computer and successfully reproduced and characterised in the lab. Yet, this is only the first step before any further industrial research and development. In this perspective, we first reviewed these few successful HTS studies, before giving some of our thoughts on what could help future HTS research reach the next stage: more efficient collaboration between experimental and computational experts *via* better data sharing, systematically including more synthesis-related metrics into HTS, and further digitalising the syntheses to ensure reproducibility and procedure shareability. These steps, summarised in Fig. 5 in their HTS context, are by no means straightforward to implement, nor are they the only solutions. But we believe the points highlighted here are exciting topics of research that could take MOFs out of the computer and bring them a few steps closer to being studied for industry-friendly systems. Finally, many of the issues raised in this perspective are applicable to the wider materials field. In this perspective, we looked at them through a MOF HTS lens to identify possible fixes – notably borrowed from other materials fields – to our own pain points. We hope this demonstrates the value of exchanging more with other research fields in finding creative solutions.

## Author contributions

D. F.-J. set the scope of the perspective. A. L. wrote the first draft of the perspective. R. B.-P. and D. M. provided insights and guidance. All authors contributed to the final version.

## Conflicts of interest

D. F.-J. and A. L. have a financial interest in the start-up company Immaterial, which is seeking to commercialise metal–organic frameworks. The remaining authors declare no competing interests.

## Supplementary Material
